# Combined use of CRP with neutrophil-to-lymphocyte ratio in differentiating between infectious and noninfectious inflammation in hemodialysis patients

**DOI:** 10.1038/s41598-023-32270-8

**Published:** 2023-04-04

**Authors:** Ilia Beberashvili, Muhammad Abu Omar, Elad Nizri, Kobi Stav, Shai Efrati

**Affiliations:** 1 Nephrology Division, Yitzhak Shamir Medical Center, Zerifin, Affiliated with the Sackler Faculty of Medicine, Tel Aviv University, 70300 Zerifin, Israel; 2 Emergency Medicine Department, Yitzhak Shamir Medical Center, Zerifin, Affiliated with the Sackler Faculty of Medicine, Tel Aviv University, Zerifin, Israel; 3 Urology Department, Yitzhak Shamir Medical Center, Zerifin, Affiliated with the Sackler Faculty of Medicine, Tel Aviv University, Zerifin, Israel

**Keywords:** Nephrology, Risk factors

## Abstract

We tested whether CRP combined with the neutrophil-to-lymphocyte ratio (NLR) optimizes the prediction of infectious inflammation in hemodialysis patients. We conducted a retrospective study of 774 (mean age 71.1 ± 12.8 years, 35% women) hemodialysis patients from our institution, hospitalized between 2007 and 2021 for various reasons, with CRP levels available at admission. Infection was defined according to the International Sepsis Definition Conference criteria. An algorithm for the optimal CRP and NLR cutoff points for predicting infection was developed based on a decision tree analysis in the training cohort (n = 620) and then tested in the validation cohort (n = 154). A CRP level above 40 mg/L (obtained as the cutoff point in predicting infections in the training group, using ROC curve analysis) predicted an infection diagnosis with a sensitivity of 75% and a specificity of 76% with an odds ratio (OR) of 9.37 (95% CI: 5.36–16.39), according to a multivariate logistic regression analysis. Whereas, CRP levels above 23 mg/L together with an NLR above 9.7 predicted an infection diagnosis with a sensitivity of 69% and a specificity of 84% with an OR of 25.59 (95% CI: 9.73–67.31). All these results were reproduced in the validation set. Combined use of CRP with NLR may lower the CRP cutoff point in distinguishing between infectious and noninfectious inflammation in hemodialysis patients.

## Introduction

C-reactive protein (CRP) is chronic inflammation marker in hemodialysis patients^1,2^. At high levels, this protein can indicate the existence of an infection^3–5^. CRP has also been widely studied as a predictor of worse cardiovascular outcomes with strong evidence showing a link between chronic inflammation and all-cause mortality^6,7^ and cardiovascular mortality^8,9^ in this population. In an attempt to define the CRP threshold levels for clinically significant inflammation in hemodialysis patients, a previous study found that CRP levels greater than 10 mg/L indicate inflammation, mainly caused by infection^10^. CRP levels above 10 mg/dl were also found associated with 1-year cardiovascular mortality in the Dialysis Outcomes and Practice Patterns Study (DOPPS) population^11^. However, the positive association with CRP was strongest for infection-related death in this population at any level of CRP^11^. High burdens of atherosclerotic cardiovascular disease and death is characteristic for hemodialysis patients with CRP levels maintained in ranges between 10 to 50 mg/L, whereas CRP levels above 50 mg/L are usually associated with acute infections and are temporary^12^. Therefore, the CRP range between 10 and 50 mg/L is very problematic in terms of predicting a potentially curable acute infection. This difficulty may lead to risk underestimation and underinvestment in the search for an acute infection, or conversely, to the unnecessary empiric use of antibiotics.

The neutrophil-to-lymphocyte ratio (NLR), a simple and available index for any type of medical institution, has been recently found to be associated with inflammation, and all-cause^13,14^ and cardiovascular mortality^14^ in hemodialysis patients. Moreover, longitudinal changes in the NLR have been shown to mimic CRP and are consequently associated with survival in hemodialysis populations^15^. We hypothesized that the combined use of CRP and NLR may lower the upper cutoff CRP point in predicting the risk of infectious inflammation and improve the performance of CRP in differentiating between infectious and noninfectious inflammation. We therefore tested whether the combined use of CRP with NLR optimizes the prediction of infectious inflammation in hemodialysis patients.

## Results

**Figure 1:**
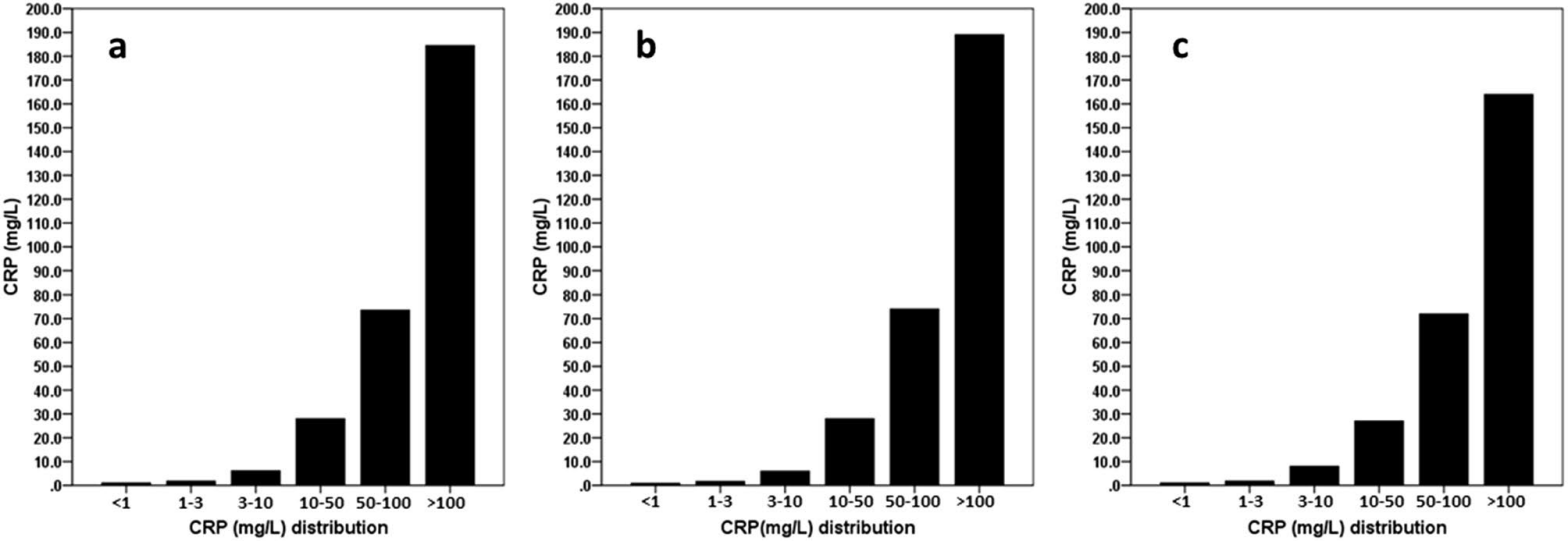
The distribution of CRP at the beginning of hospitalization in the whole cohort (**a**), in the training group (**b**) and in the validation group (**c**).

The demographic, clinical and laboratory data of the study population are shown in Table 1. The average age of the study participants was 71.1 ± 12.8 years, of which 35% were women, with a median dialysis vintage of 36 months, and 68.5% suffered from type 2 diabetes. Median CRP levels (interquartile range) at the beginning of hospitalization was 69.0 (25.5 to 157.0) mg/L. The distribution of CRP at the beginning of hospitalization was similar in the whole population (Fig. 1a), in the training group (Fig. 1b) and in the control group (Fig. 1c). Of the 578 (74.7%) patients hospitalized due to an infectious disease, 413 (71.5%) patients had a fever over 38 °C (Table 1), 193 (33.4%) patients had positive blood cultures, 68 (11.8%) patients had a positive culture from wounds, 15 (2.6%) patients with a positive urine culture, 36 (6.2%) patients with a positive sputum culture, and 28 (4.8%) patients with a positive swab for viruses (4.0% COVID-19 infection, and 0.8% influenza) (data not shown). There were no statistically significant differences in demographic, clinical and laboratory parameters between the training and validation populations except for smoking (Table 1). More smokers entered the validation group than the training group (*p* = 0.04).

**Table 1:** Demographic, clinical and laboratory data in the entire study population, and in the development and validation groups separately.

Variables	All patients	Development group	Validation group	P value
	(n = 774)	(n = 620)	(n = 154)	
Age (y)	71.1 ± 12.8	71.4 ± 12.2	70.0 ± 13.0	0.21
Sex, female n(%)	271 (35)	216 (34.8)	55 (35.7)	0.85
Vintage (months)	36.0 (12.0–60.0)	36.0 (12.0–60.0)	24.0 (12.0–60.5)	0.74
DM n(%)	530 (68.5)	424 (68.4)	106 (68.8)	0.99
Comorbidity index	6 (3–8)	5 (3–8)	6 (4–8)	0.15
Vascular access				0.44
A-V fistula n(%)	268 (34.9)	221 (35.8)	47 (31.1)	
A-V graft n(%)	31 (4.0)	26 (4.2)	5 (3.3)	
Catheter n(%)	469 (61.1)	370 (60.0)	99 (65.6)	
Kt/V	1.38 ± 0.27	1.38 ± 0.26	1.36 ± 0.30	0.53
Smoking, yes n(%)	261 (33.7)	198 (31.9)	63 (40.9)	0.04
Renal disease				0.95
Diabetic kidney n(%)	498 (64.3)	399 (64.4)	99 (64.3)	
Hypertension n(%)	131 (16.9)	105 (16.9)	26 (16.9)	
Glomerulonephritis n(%)	24 (3.1)	19 (3.1)	5 (3.2)	
ADPKD n(%)	13 (1.7)	12 (1.9)	1 (0.6)	
Obstruction n(%)	13 (1.7)	10 (1.6)	3 (1.9)	
Others n(%)	95 (12.3)	74 (11.9)	20 (13.0)	
BMI (kg/m^2^)	26.7 ± 5.9	26.7 ± 6.1	26.8 ± 5.4	0.92
Hb (g/dl)	10.6 ± 1.9	10.6 ± 1.9	10.3 ± 1.9	0.07
WBC (× 10^3^/mm^3^)	9.8 (6.8–14.0)	9.8 (6.8–14.0)	9.6 (6.9–14.1)	0.99
PLT (× 10^3^/mm^3^)	189.5 (144.0–253.0)	189.0 (145.0–252.0)	194.5 (137.8–253.5)	0.97
NLR	8.77 (4.70–15.92)	8.65 (4.70–15.70)	9.15 (4.40–16.2)	0.97
Albumin (g/dl)	3.25 ± 0.6	3.26 ± 0.6	3.22 ± 0.6	0.48
Creatinine (mg/dl)	5.59 ± 2.1	5.54 ± 2.0	5.77 ± 2.5	0.24
Uric acid (mg/dl)	5.1 ± 1.7	5.1 ± 1.7	5.1 ± 1.6	0.72
CRP (mg/L)	69.0 (25.5–157.0)	65.0 (24.0–164.0)	82.0 (33.5–146.0)	0.24
Ferritin (ng/ml)	711.0 (325.0–1259.0)	661.5 (312.0–1196.5)	905.0 (369.0–2421.0)	0.14
Fever n(%)	413 (53.4)	324 (52.3)	89 (57.8)	0.24
Infection n(%)	578 (74.7)	462 (74.5)	116 (75.3)	0.92
CVD hospitalization* n(%)	96 (12.4)	81 (13.1)	15 (9.7)	0.34

Normally distributed continuous variables are expressed as means (SDs), as medians (interquartile ranges) for non–normally distributed data, and categorical variables are expressed as percentages.

*Cardiovascular disease (CVD) related hospitalization was defined as hospitalization due to coronary heart disease, heart failure, stroke, or peripheral vascular disease.

*DM*, diabetes mellitus, *A-V*, arterial-venous, *ADPKD*, autosomal-dominant polycystic kidney disease, *BMI*, body mass index, *Hb*, hemoglobin, *WBC*, white blood cells, *PLT*, platelets, *NLR*, neutrophil-to-lymphocyte ratio, *CRP*, C-reactive protein, *CVD*, cardiovascular disease.

**Figure 2:**
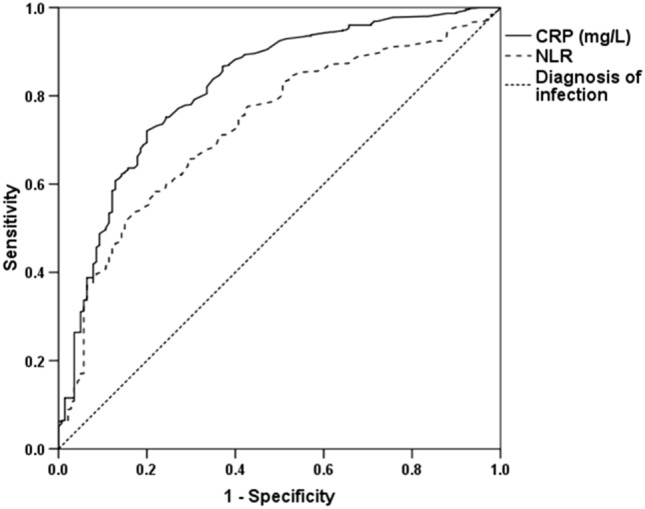
Receiver operating characteristic curve of baseline CRP and NLR in the training cohort (n = 620); fraction of true-positive (sensitivity) and false-positive results (1-specificity) for CRP and NLR as a marker of the diagnosis of infection. AUC for CRP-0.82 (0.78–0.86), P < 0.001, cut-off of ≥ 40 mg/L predicts diagnosis of infection with a sensitivity of 75% and a specificity of 76%; and AUC for NLR-0.73 (0.69–0.78), *P* < 0.001, cutoff of ≥ 7.41 predicts an infection diagnosis with a sensitivity of 67% and a specificity of 67%.

Cutoff points of CRP and NLR in predicting an infection diagnosis were evaluated using the area under the ROC curves (AUCs) in the training group (see Fig. 2). AUC for CRP was 0.82 (0.78–0.86), *P* < 0.001, with a cutoff of ≥ 40 mg/L in predicting an infection diagnosis with a sensitivity of 75% and a specificity of 76%; the AUC for NLR was 0.73 (0.69–0.78). *P* < 0.001, with a cutoff of ≥ 7.41 in predicting an infection diagnosis with a sensitivity of 67% and a specificity of 67%.

**Figure 3:**
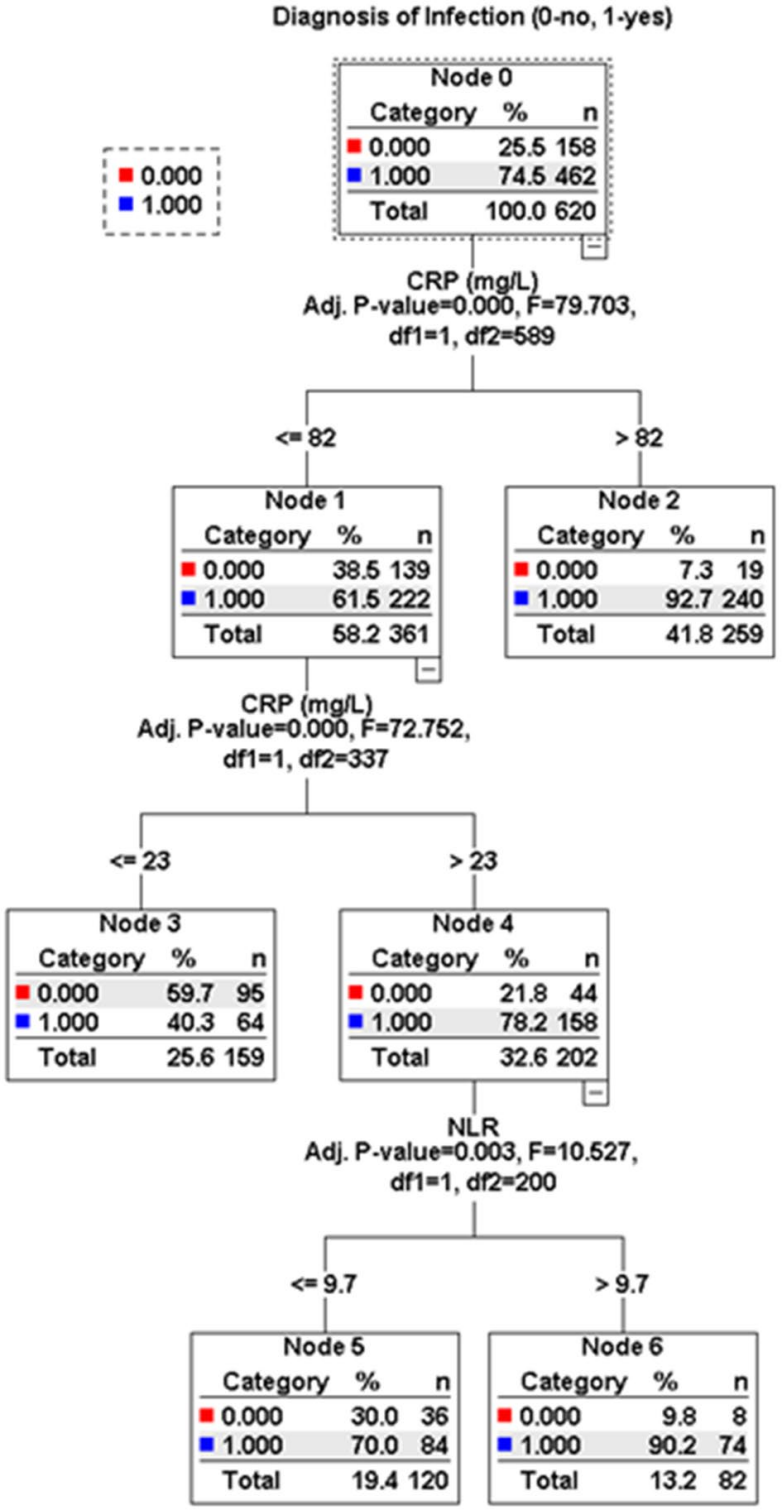
Algorithm for the optimal cutoff points of CRP (mg/L) and NLR for predicting infection based on a random forest analysis in the training cohort (n = 620).

Next, using the decision tree algorithm (QUEST method), we found cutoff points for CRP and NLR for their combined use in predicting an infection (Fig. 3). According to this algorithm, if CRP is over 82 mg/L, the probability of infection is very high (above 90%) and an infection source must be sought. If CRP is in the range of 23–82 mg/L, but with an NLR level above 9.7, there is a very high probability (over 90%) of infection. The algorithm was not helpful for patients with NLR less than 9.7 and CRP in the range 23–82 mg/L, because the probability of infectious inflammation still prevailed over noninfectious inflammation (Fig. 3). However, combining the use of CRP and NLR helped us lower the upper CRP cutoff point from 40 to 23 in the training group, in differentiating between infectious and noninfectious inflammation.

We further tested the AUC of the combined use of NLR and CRP in predicting infection and compared it to separate AUCs of CRP above 40 mg/L and NLR above 7.41 (cutoffs for CRP and NLR in the training group for predicting infectious inflammation obtained by ROC analyses, see Fig. 2) in the training and validation groups (Table 2). The AUC for the combined use of CRP and NLR was not inferior to the AUC for CRP and was higher than the AUC for NLR in both training and validation groups. The sensitivity of the test for predicting infection decreased slightly in cases of combined use of CRP and NLR compared to CRP above 40 mg/L but its specificity was noticeably higher than CRP above 40 mg/L. The combined use of CRP and NLR had the highest likelihood ratio (= 4.18) and CRP above 40 mg/L had the lowest likelihood ratio (= 0.33) in the training group. This means that infectious-related inflammation is 4.18 times more likely in MHD patients with CRP > 82 mg/dl or CRP in the range of 23–82 mg/L with NLR above 9.7 than in MHD patients with values lower than these cutoff points. Furthermore, MHD patients with CRP below 40 mg/L have a sevenfold decrease in the odds of having an infection than MHD patients with CRP above 40 mg/L. Whereas in MHD patients with CRP less than 23 mg/L or CRP in the range of 23–82 mg/L with NLR below 9.7, there is about a sixfold decrease in the likelihood of infection compared to values higher than these cutoff points in the training group. All these results were reproduced in the validation set (Table 2).

**Table 2:** Comparing the AUC of CRP, NLR and their combination in predicting infection in the development and validation groups.

Variable	CRP ≥ 40 mg/L	NLR ≥ 7.41	Combined CRP and NLR	*P* value*
Development cohort (n = 620)				
AUC (95% CI)	0.75 (0.71–0.80)	0.68 (0.63–0.73)	0.76 (0.72–0.81)	P_1_ = 0.57
*P*	< 0.001	< 0.001	< 0.001	P_2_ < 0.001
Sensitivity (%)	75	68	69	
Specificity (%)	76	68	84	
+ LR	3.08	2.06	4.18	
− LR	0.33	0.5	0.37	
Validation cohort (n = 154)				
AUC (95% CI)	0.73 (0.63–0.83)	0.62 (0.51–0.73)	0.75 (0.66–0.84)	P_1_ = 0.62
*P*	< 0.001	0.03	< 0.001	P_2_ = 0.01
Sensitivity (%)	82	66	72	
Specificity (%)	64	58	78	
+ LR	2.26	1.58	3.24	
− LR	0.29	0.6	0.36	

*The receiver operating characteristic (ROC) curves in the development and validation groups were compared by DeLong’s method.

P_1_-*P* value of combined CRP and NLR vs CRP ≥ 40 mg/L; and.

P_2_-*P* value of combined CRP and NLR vs NLR ≥ 7.41.

Table 3 shows demographic, clinical and laboratory data of the study participants in the development group stratified by CRP, NLR levels and their combined use. Patients with higher inflammatory markers (according to CRP, NLR or their combination) had more anemia, more fever and number of infections and lower albumin as expected.

**Table 3:** Demographic, clinical and laboratory data of the study participants in the development group (n = 620) stratified by CRP, NLR levels and their combined use.

Variables	CRP		NLR		CRP-NLR comb^#^	
	< 40 mg/L	≥ 40 mg/L	< 7.41	≥ 7.41	Low	High
	(n = 220)	(n = 400)	(n = 253)	(n = 367)	(n = 258)	(n = 362)
Age (y)	70.1 ± 12.8	71.8 ± 12.8	71.1 ± 12.9	71.6 ± 12.6	71.0 ± 12.9	71.3 ± 12.8
Sex, female n(%)	75 (34.1)	129 (32.3)	96 (37.9)	120 (32.7)	91 (35.3)	113 (31.2)
Vintage (months)	31 (11–60)	36 (12–60)	36 (13–62)	34 (12–60)	36 (12–65)	31 (12–60)
DM n(%)	151 (68.6)	258 (64.5)	167 (66.0)	257 (70.0)	169 (65.5)	240 (66.2)
Comorbidity index	6 (3–8)	5 (3–9)	6 (3–8)	5 (3–8)	6 (3–8)	5 (3–8)
Vascular access						
A-V fistula n(%)	93 (42.3)	121 (30.3)	97 (38.3)	124 (33.8)	106 (41.1)	107 (29.6)
A-V graft n(%)	10 (4.5)	15 (3.8)	9 (3.6)	17 (4.6)	10 (3.9)	15 (4.1)
Catheter n(%)	117 (53.2)	232 (58.0)	145 (57.5)	224 (61.4)	141 (54.9)	209 (57.7)
Kt/V	1.39 ± 0.27	1.38 ± 0.26	1.38 ± 0.25	1.38 ± 0.27	1.40 ± 0.26	1.37 ± 0.26
Smoking, yes n(%)	70 (31.8)	121 (30.2)	76 (30.0)	122 (33.2)	81 (31.4)	110 (30.4)
Renal disease						
Diabetic kidney n(%)	142 (64.5)	242 (60.5)	164 (64.6)	236 (64.3)	160 (61.8)	224 (61.9)
Hypertension n(%)	35 (15.9)	62 (15.5)	43 (17.0)	62 (16.5)	49 (18.2)	50 (13.8)
Glomerulonephritis n(%)	9 (4.1)	10 (2.5)	8 (3.2)	11 (3.0)	12 (4.7)	7 (1.9)
ADPKD n(%)	13 (1.7)	4 (1.0)	7 (2.8)	5 (1.4)	7 (2.7)	4 (1.1)
Obstruction n(%)	7 (3.2)	2 (0.5)	4 (1.6)	6 (1.6)	6 (2.3)	2 (0.6)
Others n(%)	21 (9.5)	51 (12.8)	27 (10.7)	47 (12.8)	26 (10.1)	46 (12.7)
BMI (kg/m^2^)	27.1 ± 6.1	26.5 ± 6.2	26.8 ± 5.6	26.7 ± 6.4	26.8 ± 5.8	26.7 ± 6.4
Hb (g/dl)	11.2 ± 1.8	10.2 ± 1.8^c^	10.9 ± 1.9	10.4 ± 1.8^b^	11.1 ± 1.9	10.3 ± 1.8^c^
WBC (× 10^3^/mm^3^)	7.9 (6.1–11.2)	11.8 (7.8–15.3)^c^	7.7 (5.7–10.1)	11.8 (8.4–15.4)^c^	7.9 (6.0–10.7)	12 (8.4–16)^c^
PLT (× 10^3^/mm^3^)	178(146–225)	196(143–266)	181(143–232)	196(145–262)	177(146–228)	200(144–268)^b^
NLR	5.9 (3.5–9.6)	11.7 (6.7–20. 0)^c^	4.2 (2.9–5.5)	13.8 (9.8–22.0)^c^	5.4 (3.27.7)	14 (9.2–22.0)^c^
Albumin (g/dl)	3.53 ± 0.5	3.01 ± 0.5^c^	3.45 ± 0.5	3.13 ± 0.6^c^	3.49 ± 0.5	3.06 ± 0.5^c^
Creatinine (mg/dl)	5.65 ± 1.9	5.45 ± 2.0	5.67 ± 2.0	5.46 ± 2.0	5.58 ± 1.9	5.48 ± 2.0
Uric acid (mg/dl)	5.0 ± 1.5	5.2 ± 1.9	4.9 ± 1.6	4.9 ± 1.6	4.9 ± 1.5	5.2 ± 1.9^a^
CRP (mg/L)	16 (7–28)	129 (73–209)^c^	33 (11–80)	104 (41–202)^c^	20 (8–37)	145 (83–222)^c^
Ferritin (ng/ml)	503(288–739)	839(399–1949)^b^	585(312–987)	816(275–1295)	529(228–919)	850(409–2046)^b^
Fever n(%)	77 (35.0)	239 (59.8)^c^	100 (39.5)	224 (61.0)^c^	95 (36.3)	221 (61.0)^c^
Infection n(%)	114 (51.3)	337 (84.3)^c^	148 (58.5)	314 (85.6)^c^	141 (54.7)	310 (85.6)^c^
CVD hospitalization* n(%)	61 (27.7)	16 (4.0)^c^	65 (25.7)	16 (4.3)^c^	68 (26.4)	9 (2.5)^c^

Normally distributed continuous variables are expressed as means (SDs), as medians (interquartile ranges) for non–normally distributed data, and categorical variables are expressed as percentages.

The cutoffs for CRP or NLR (40 mg/L and 7.41, respectively) were obtained by ROC analyses (see Fig. 2).

^#^A low level of the combined use of CRP and NLR was determined if the CRP level was less than 23 mg/L, or it was between 23 and 82 mg/L but with an NLR less than 9.7. A high level of the combined use of CRP and NLR was determined if the CRP level was above 82 mg/L, or it was between 23 and 82 mg/L but with an NLR above 9.7.

*Cardiovascular disease (CVD) related hospitalization was defined as hospitalization due to coronary heart disease, heart failure, stroke, or peripheral vascular disease.

^a^*p* < 0.05.

^b^*p* < 0.01.

^c^*p* < 0.001.

*NLR*, neutrophil-to-lymphocyte ratio, *CRP*, C-reactive protein, *CRP-NLRcomb*, combination of CRP and NLR, *DM*, diabetes mellitus, *A-V*, arterial-venous, *ADPKD*, autosomal-dominant polycystic kidney disease, *BMI*, body mass index, *Hb*, hemoglobin, *WBC*, white blood cells, *PLT*, platelets, *CVD*, cardiovascular disease.

Table 4 shows crude and adjusted odds for predicting infection in MDH patients in both training and validation cohorts, using cutoffs for CRP, NLR and their combination obtained by ROC and the random decision tree analyses (see Figs. 2 and 3). ORs were noticeably higher in predicting infection with the combined use of CRP and NLR than with CRP or NLR alone in both univariate and multivariate analyses (adjusted for age, sex, dialysis vintage, DM status, vascular access type, Kt/V, comorbidity index, underlying kidney disease and smoking).

**Table 4:** Crude and adjusted^a^ odds for infection prediction grouped by CRP, NLR levels^b^ and their combined use according to univariate and multivariate logistic regression analyses.

Variable		Development cohort (n = 620)		Validation cohort (n = 154)	
		OR (95% CI)	*P*	OR (95% CI)	*P*
CRP ≥ 40 mg/L	Crude	9.22 (5.93–14.32)	< 0.001	7.83 (3.42–17.94)	< 0.001
	Adjusted	9.37 (5.36–16.39)	< 0.001	6.62 (2.31–18.91)	< 0.001
NLR ≥ 7.41	Crude	4.20 (2.63–5.20)	< 0.001	2.63 (1.22–5.53)	0.01
	Adjusted	3.93 (2.50–6.18)	< 0.001	3.42 (1.28–9.17)	0.01
Combined use of CRP and NLR					
CRP ≤ 23 mg/L (n = 167)	Crude	Ref		Ref	
	Adjusted	Ref		Ref	
CRP 23–82 mg/L, NLR ≤ 9.7 (n = 97)	Crude	3.86 (2.27–6.54)	< 0.001	4.64 (1.56–13.81)	< 0.001
	Adjusted	4.83 (2.53–9.20)	< 0.001	3.90 (0.89–17.17)	0.07
CRP 23–82 mg/L, NLR > 9.7 (n = 143)	Crude	15.41 (6.63–15.80)	< 0.001	7.60 (1.73–33.34)	0.007
	Adjusted	25.59 (9.73–67.31)	< 0.001	14.64 (1.88–114.3)	0.01
CRP > 82 mg/L (n = 334)	Crude	21.50 (11.74–39.38)	< 0.001	26.60 (8.12–87.19)	< 0.001
	Adjusted	29.92 (13.01–68.79)	< 0.001	50.00 (8.67–288.3)	< 0.001

^a^Adjusted for age, sex, dialysis vintage, DM status, vascular access type, Kt/V, comorbidity index, underlying kidney disease, smoking, hemoglobin and albumin.

^b^The cutoffs for CRP or NLR (40 mg/L and 7.41, respectively) were obtained by ROC analyses (see Fig. 2).

**Figure 4:**
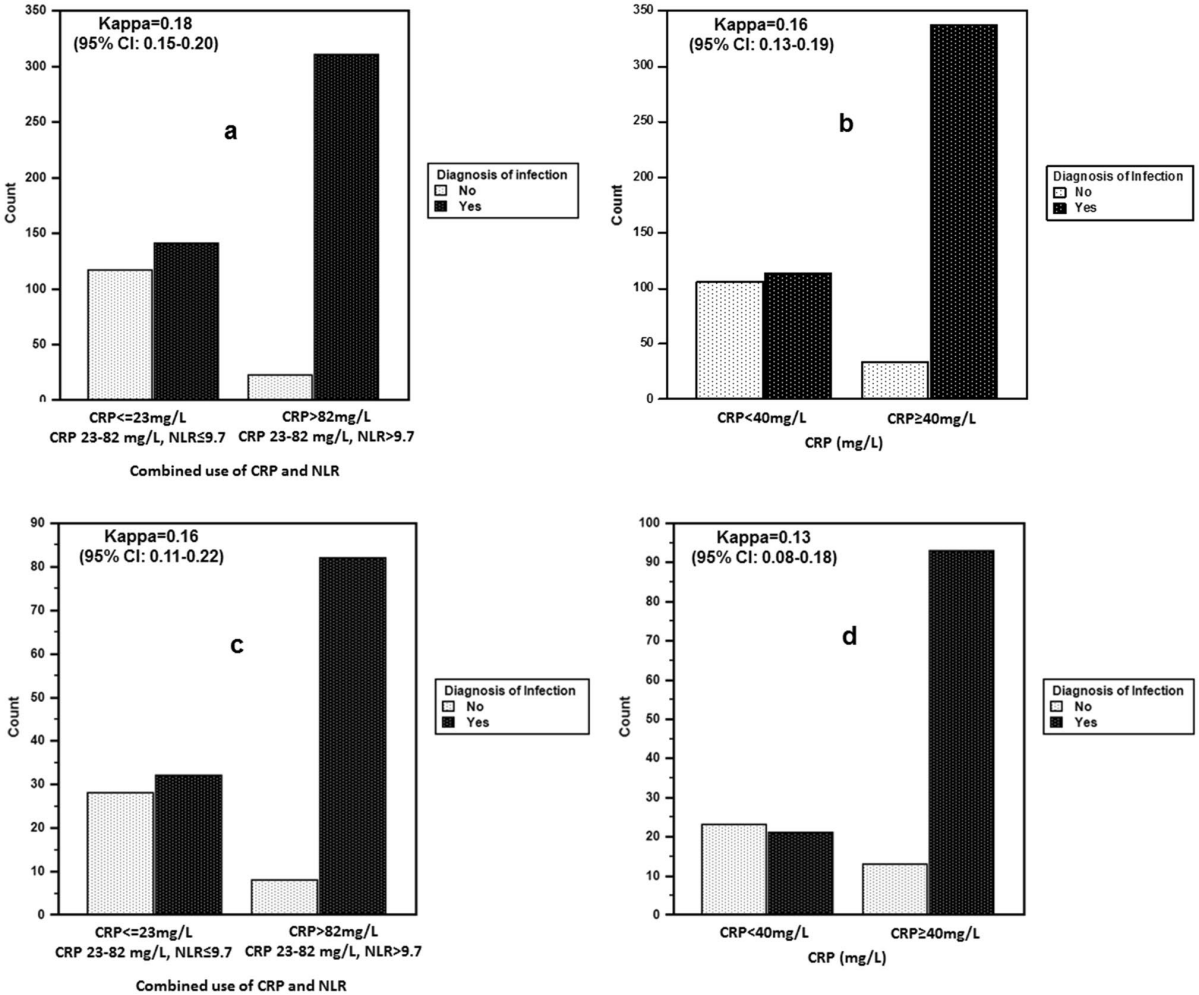
Proportion of agreement to disagreement of the combined use of CRP with NLR and CRP ≥ 40 mg/L, based on kappa statistics in the training (**a** and **b**) and validation (**c** and **d**) groups of MHD patients.

Finally, the agreement between predicting infection based on CRP values alone or combining CRP-NLR at the beginning of hospitalization and a clinical diagnosis of infection made during hospitalization was calculated in the training and validation groups using the kappa test for inter-rater agreement (Fig. 4). Although the weighted κ coefficient was higher in cases of using criteria based on a combination of CRP with NLR compared to criteria based on CRP values alone, it did not exceed 0.2 in either the training group (Fig. 4a and b, respectively) or the validation group (Fig. 4c and d, respectively). A possible explanation is that CRP below 23 mg/L, or CRP in the range between 23 and 82 mg/L with an NLR below 9.7, as well as CRP below 40 mg/L (using criteria based on CRP only), are very problematic in terms of predicting infection due to a significant prevalence of infectious inflammation in these groups (Fig. 4).

## Discussion

In this study, we found that the combined use of CRP and NLR may lower the cutoff of CRP in distinguishing between infectious and noninfectious inflammation in hemodialysis patients without losing the classification model’s performance (area under the receiver operating characteristics curve). Specifically, we built an algorithm using CRP and NLR for this purpose in a training population and validated it in a validation population.

The relationship between CRP and its cutoff level for early detection of active infections has been extensively studied in different populations so far^16–19^. CRP levels vary greatly with age, sex and race. Additionally, in some noninfectious "metabolic inflammatory" conditions (such as smoking, uremia, cardiac ischemia) CRP levels can rise to 2–10 mg/L, while mild to moderate disorders (such as an uncomplicated skin infection, urinary tract infection or pneumonia) can raise CRP to 50–100 mg/L within six hours^16^. In a prospective study of hospitalized patients aged 70 and over, it was found that the cutoff CRP value of 60 mg/L had the best combination of sensitivity (81%) and specificity (92%) for predicting bacterial infections^17^. In early detecting of bacterial infection in febrile patients, CRP performance was inferior to procalcitonin with an AUC of 0.693 (0.639–0.742), a cutoff value of 73.8 mg/L, a sensitivity of 62%, and a specificity of 72%, in 326 patients admitted to the Department of Infectious Diseases in West China Hospital^18^. The specificity and diagnostic accuracy of CRP were lower than that of procalcitonin also in differentiating bacterial infections from disease flare-ups in patients with systemic rheumatic diseases in a meta-analysis of eight studies, including 668 patients^19^. Although the above studies investigated CRP levels as a predictor of bacterial infection, it should be realized that CRP levels cannot distinguish between types of infection, because infections in general cause CRP levels to rise, and not the type of infection^20^. In dialysis patients compared to other populations, it is more complicated to interpret high levels of CRP due to a high incidence of chronic inflammation in the uremic milieu^1,6,7^. In a prospective longitudinal observational study by Snaedal et al.^21^, only 13% of a cohort of 254 prevalent hemodialysis patients from six dialysis units from Sweden had constantly low CRP levels (less than 5 mg/L), whereas 19% had CRP values greater than 10 mg/L, and 68% of patients had fluctuating values depending on age, sex, comorbidity, vintage, and access type. While the diagnostic performance of serum CRP in 68 hospitalized hemodialysis patients indicating severe infections and sepsis with a CRP cutoff of 11.2 mg/L, yielded a sensitivity of 89%, the specificity was only 48%^22^. CRP levels above 100 mg/L were found to have a 100% positive predictive value and a 94% negative predictive value for the diagnosis of sepsis in 802 hemodialysis patients^5^. Our finding of a high prevalence of infection across all CRP ranges is in line with a study by Bazeley et al.^11^ exploring CRP based prediction of 1-year mortality in the DOPPS population. From our point of view, the significant presence of infection in all CRP ranges is the main reason for low Kappa scores for the inter-rater agreement observed in our study between the CRP-NLR combination predicted infection and a clinical diagnosis of infection. Of note, a CRP cutoff point of 40 mg/L that we obtained in our training population is very close to the cut-off point of 50 mg/L that is accepted in recent literature^12^ for distinguishing infectious inflammation from noninfectious inflammation in chronic kidney disease population.

Integrated use of NLR and CRP in differentiating infectious and noninfectious inflammation was not done in previous studies and our study is the first of its kind. However, there have been attempts to combine CRP and NLR for other purposes, such as for predicting the prognosis in patients with gastric cancer^23^, non-small-cell lung cancer^24^, and in acute myocardial infarction patients undergoing percutaneous coronary intervention^25^, in patients with COVID-19 pneumonia^26^ and for diagnosing spontaneous bacterial peritonitis in cirrhotic patients^27^. We found only the one small study performed on 100 hemodialysis patients that investigated the role of NLR, CRP and procalcitonin and their combination with a retrospective case–control design for the diagnosis of pulmonary infection^28^, but without the possibility to draw clear conclusions. There are however, data in the literature of the combined use of CRP with procalcitonin, another infection marker, in dialysis patients, to differentiate between infectious and noninfectious inflammations^22,29^. The concomitant elevations in procalcitonin and CRP are hypothesized to be more sensitive in evaluating inflammation in hemodialysis patients than each marker separately^29^. While procalcitonin was found as a useful marker for diagnosis of bacterial infections in hemodialysis patients with a cutoff value of 1.5 ng/ml^30^, its value in making a diagnosis and predicting long-term prognosis remains doubtful in peritoneal dialysis (PD) patients with PD-related peritonitis^31^. The previous study, specifying the cutoff values of both procalcitonin and CRP for early detection of infection in hemodialysis patients, was able to lower the cutoff point of CRP to 19.15 mg/L, through the combined use of procalcitonin and CRP^5^. However, elevated CRP but not raised procalcitonin was found to be associated with increased inflammation and mortality in a two-year prospective study in a hospital-based cohort of high-risk hemodialysis patients^32^. The combination of high procalcitonin and CRP was no more predictive of mortality than high CRP alone in this study. Overall, based on the available data, in terms of cost-effectiveness the combination of CRP and NLR can be similar to the combination of CRP with procalcitonin. Still, to combine procalcitonin with CRP for the early detection of infectious inflammation, a blood procalcitonin level test is required, which is not available everywhere. In this respect, the use of NLR is much more convenient, available in all medical institutions and its level above 9.7 allows predicting infectious inflammation in hemodialysis patients with a CRP level above 23 mg/L. Properties of CRP as an acute phase protein^33^ and NLR indicate the balance between innate immune responses (neutrophils) and adaptive immune responses (lymphocytes)^34^. This makes their combination a good indicator of infection and inflammation and constitute the biological basis of our study.

Our study has several limitations. First, the infection rate was reported as 5.7 episodes per 1000 days of dialysis^35^ in 433 dialysis patients at a single hospital-based dialysis center and its satellites over a 9-year period (2412 episodes of bacterial or fungal infections, 424 700 days of dialysis days). A population-based cohort study in Denmark showed that the incidence of bacteremia was 13.7 per 100 person years in hemodialysis patients^36^. The 774 hemodialysis patients included in our study with 578 infections over a 15-year period could correspond to an infection rate of 4.98 per 100 person-years (0.14 episodes per 1000 days of dialysis) which is lower than that reported in this population^35,36^. However, our study was not designed to assess the burden of infection in hemodialysis patients. To answer the research question, our cohort was retrospectively selected from hospitalized hemodialysis patients based on available CRP and NLR levels on admission. In addition, the hemodialysis patients treated for infection on an ambulatory basis were not taken into account due to the lack of the possibility to obtain the study measurements. All of this explains the aforementioned gap between the reported infection rates and our data and in fact constitutes a selection bias typical of retrospective studies.

. Second, in the absence of a gold standard for the diagnosis of infection, there may be some misclassifications of the infection status in our study. However, all similar studies use the definition of infection we used with an unavoidable methodological limitation. To overcome this limitation, all ambiguous cases were excluded from our study. Third, our study represents a single center, so the results cannot be generalized to all dialysis populations. The cutoff points of CRP and NLR should be determined to construct algorithms similar to ours in more representative populations by geographic location, races, and different ages in large epidemiological studies. Further, because of the retrospective design, we were unable to obtain several relevant markers for the diagnosis of infection such as procalcitonin.

In conclusion, we have shown that the combined use of routine laboratory tests such as NLR and CRP may be used to predict infection in maintenance hemodialysis patients, and can help in their early management to reduce the incidence of subsequent complications. Specifically, combined use of CRP with NLR may lower the CRP cutoff point in distinguishing between infectious and noninfectious inflammation in hemodialysis patients. Future large-scale studies are needed to confirm our results and apply this method to daily clinical work to improve the performance of CRP in predicting infectious diseases in maintenance hemodialysis patients.

## Methods

### Patients

We conducted a retrospective cross-sectional study of 774 hemodialysis patients from our institution, hospitalized between 2007 and 2021 for various reasons, with CRP levels available at admission. This study was approved by our local institutional ethics committee (Helsinki Committee, Shamir Medical Center). All methods were performed in accordance with the relevant guidelines and regulations. We were exempted from needing a written informed consent due to the study’s retrospective design.

The database included patient demographic data, course of hospitalization, regular medications and medications given during hospitalization, laboratory values, dialysis treatment records, and comorbidities. We included in the study males and females over the age of 18 with ESKD who received maintenance hemodialysis treatments and were hospitalized for any reason. Patients receiving regular treatment with steroids over 10 mg of prednisone per day (or other steroids in equivalent doses), receiving treatment with immunosuppressive drugs of any type, those suffering from symptoms more than three days (such as fever, chills and sweating, cough, sore throat, shortness of breath, nasal congestion, dysuria, diarrhea, vomiting, abdominal pain) before arriving at the emergency room or those who reported a bacterial infection four weeks before their admission were excluded from the study. All patients underwent regular dialysis via their vascular access for 4–5 h three times per week, at a blood flow rate of 250–300 ml/min and a dialysis solution flow rate of 500 ml/min. Dialysis treatments were performed with biocompatible dialyzer membranes with surface areas of 1.4–1.8 m^2^.

Of the study participants, 620 patients were randomly selected for a training group for the development of a diagnostic algorithm to distinguish between infectious and non-infectious inflammation, and the remaining 154 patients were used to validate the final constructed algorithm.

### Comorbidity index and clinical outcomes

We determined the comorbidity index, which was recently developed by Liu et al.^37^ and validated specifically for populations of patients on dialysis, as a measure of comorbid conditions. The infectious status of the patients was defined according to the International Sepsis Definition Conference (ISDC)^38^:

1. Definite infection—patients with a definite infection established according to clinical and microbiological criteria.
2. Probable infection—patients with clinical manifestations of an infection plus radiological evidence without a positive blood culture.
3. Possible infection—patients with clinical features of an infection without established microbiological or radiological evidence.
4. Non-infection—defined as no clinical, microbiological or radiological evidence of bacterial or viral infections.

We also obtained the final diagnosis of infection or non-infection as made by the treating clinicians. All radiological findings were confirmed by radiologists. Cases with clinical ambiguity that made it difficult to classify as an infectious condition were excluded.

### Laboratory evaluation

Blood samples were obtained from nonfasting patients on a midweek day predialysis, with the exception of postdialysis serum urea nitrogen to calculate urea kinetics. Albumin was measured using the bromocresol green method. All biochemical analyses, including CBC, creatinine, urea, albumin, uric acid, and ferritin, were measured by an automatic analyzer. Additionally, serum high–sensitivity CRP was measured by a turbidimetric immunoassay.

### Statistical analyses

Normally distributed data are expressed as means ± SDs, as medians and interquartile ranges (quartiles 1–3) for variables with skewed distributions, or as frequencies for categorical variables.

Normally distributed continuous variables were compared between the two groups using a two–sided t test, with chi-squared tests used for categorical variables, and nonparametric Mann–Whitney U tests were used for non-normally distributed continuous variables.

The cutoff for the most accurate discrimination of infection prediction risk for CRP and NLR were derived using standard receiver operating characteristic (ROC) curves. These cutoff points were used to calculate the sensitivity and specificity of CRP and NLR in predicting infection. The ROC curves in the development and validation groups were compared by DeLong’s method^39^. To determine the posttest probability, the positive likelihood ratio (LR +) and negative likelihood ratio (LR −) were calculated as follows:

$${\text{LR}} + = {\text{ sensitivity}}/\left( {{1}{-}{\text{specificity}}} \right)$$

and

$${\text{LR}} - = \left( {{1}{-}{\text{sensitivity}}} \right)/{\text{specificity}}.$$

A multiple logistic regression analysis was used to provide adjusted odds ratios and 95% confidence intervals (CI) for CRP and NLR as independent risk factors. All variables that were hypothesized on theoretical grounds or were shown in previous studies to be confounders of the association between CRP, NLR and infectious inflammation were included as confounders in our multivariable models. Therefore, all models were adjusted for age, sex, dialysis vintage, DM status, vascular access type, Kt/V, comorbidity index, underlying kidney disease and smoking.

The decision tree algorithm, particularly the QUEST (quick, unbiased, efficient statistical tree) method, was used to evaluate the NLR and CRP levels in predicting infection. QUEST is a binary-split decision tree algorithm for classification and data mining that supports univariate and linear hybrid segmentation^40^.

The agreement between predicting infection based on CRP values alone or the combined use of CRP and NLR at the beginning of hospitalization and a clinical diagnosis of infection made during hospitalization, was calculated using the kappa test for inter-rater agreement.

All statistical analyses were performed using SPSS software, version 18.0 (IBM SPSS, Chicago, IL).

## Data Availability

The data that support the findings of this study are available from the corresponding author upon reasonable request.
